# The Draft Genome Sequence of a New Land-Hopper *Platorchestia hallaensis*

**DOI:** 10.3389/fgene.2020.621301

**Published:** 2021-01-11

**Authors:** Ajit Kumar Patra, Oksung Chung, Ji Yong Yoo, Sang Ho Baek, Tae Won Jung, Min Seop Kim, Moon Geun Yoon, Youngik Yang, Jeong-Hyeon Choi

**Affiliations:** ^1^Department of Life Science, Ewha Womans University, Seoul, South Korea; ^2^Clinomics Inc., Ulsan, South Korea; ^3^Marine Bio Resources and Information Center, National Marine Biodiversity Institute of Korea, Seocheon, South Korea; ^4^Research Center for Endangered Species, National Institute of Ecology, Yeongyang, South Korea; ^5^Department of Ecology and Conservation, National Marine Biodiversity Institute of Korea, Seocheon, South Korea; ^6^Department of Applied Research, National Marine Biodiversity Institute of Korea, Seocheon, South Korea

**Keywords:** land hopper, *Platorchestia*, *Talitridae*, draft genome, next generation sequencing

## Introduction

Unlike the limited geographical distribution of most of the genera within the family Talitridae (Crustacea, Amphipoda) (Wildish, 1988), the genus *Platorchestia* is distributed in each continent except for the polar regions (Wildish and Radulovici, 2019). But several species of *Platorchestia* are predominantly found in the North Pacific Ocean region. Most talitrids live in the tidal zones near to the estuary or near to the seashore. However, larger number of *Platorchestia* species are found in various ecotypes such as marine or estuarine wrack, terrestrial leaf litter, freshwater, and saltwater marsh, and occasionally found in caves and driftwoods (Wildish and Radulovici, 2019). These ecosystems share common characteristics such as moisture, but different salinity and temperature condition. It was hypothesized that *Platorchestia* originated on the South-easterly coast of Laurasia, roughly and would evolve along separate and independent evolutionary lines in the Atlantic and Pacific (Wildish and Radulovici, 2019). In this study, *Platorchestia hallaensis* was found in the leaf litter of the cave in Halla mountain of South Korea.

Currently the genus *Platorchestia* consists of 18 morphologically characterized species (World Register of Marine Species) as of 2 September 2020. Usually, the mitochondrial cytochrome c oxidase subunit 1 (COI) gene sequence is primarily used as a DNA barcode for molecular identification, taxonomy, phylogeny, and phylogeographical studies of most Platorchestia species. The molecular database for DNA barcodes [Barcode of Life Data System (Ratnasingham and Hebert, 2007)] was inquired on 2 September 2020 and we found 469 sequences for 11 *Platorchestia* species. These 11 species had 25 clustered COI sequences in cohesive genetic groups also known as Barcode Index Numbers (BINs) indicating shared genetic relationships among *Platorchestia* species (Ratnasingham and Hebert, 2007). Due to identical morphological features, taxonomists have struggled to distinguish between genetically different although closely related *Platorchestia* species, e.g., *P. platensis* and *P. monodi* (Stock, 1996; Serejo and Lowry, 2008; Radulovici, 2012). The taxonomical identification process of *Platorchestia* species remains inadequate and insufficient which requires multiple integrated methods to distinguish species within the genus from the wide geographical regions.

An analysis based on single or handful genetic markers can hardly reflect species divergence at the genome scale. Currently, there are only two complete mitochondrial genome sequences of Platorchestia: *P. parapacifica* and *P. japonica* (Yang et al., 2017). Among 228 families consisting of more than 10,200 species in the order Amphipoda, only four genomes have been studied (Zeng et al., 2011; Rivarola-Duarte et al., 2014; Poynton et al., 2018; Patra et al., 2020), which includes the genome of talitrid *Trinorchestia longiramus* (Patra et al., 2020). Thus, we sequenced and assembled a reference genome for this *Platorchestia* species with an aim to set up a genetic platform for studying their taxonomical identification, divergence, and evolutionary history.

In this study, we present the first draft genome of *Platorchestia hallaensis* using the Illumina HiSeq 2500 platform. The genome size of *P. hallaensis* was estimated *in silico* at ~1.43 Gb. The draft genome was assembled into 39,877 scaffolds (N50 = 86.5 kb), with a total size of 1.18 Gb and 84.7% genome completeness by BUSCO. Structural gene annotation predicted 19,780 genes (21,556 transcripts) with 86.7% transcriptome completeness by BUSCO. We functionally annotated 12,237 genes with known databases. The comparative genomics among 13 arthropod species identified unique gene clusters in *P. hallaensis*, as well as contracted and expanded gene clusters in 13 arthropod species and their ancestors. A phylogenetic analysis with 13 arthropod species suggested that *P. hallaensis* diverged from *T*. *longiramus* (Patra et al., 2020) during the Middle Cenozoic era. This talitrid genome will play a key role in further studies on the molecular mechanisms for adaptation of talitrids in diverse habitats and genomic variation across amphipods.

## Materials and Methods

### Sample Collection and Extraction of DNA and RNA

*P. hallaensis* samples were collected from the cave (33°30′6.03″N, 126°46′17.87″E) of South Korea. They were captured by hand from dark, humid place under rocks or fallen leaves in the cave. Samples were preserved immediately in 95% ethanol for genome sequencing or stored in liquid nitrogen for RNA extraction. DNA was extracted from a pool of the whole body of seven adult individuals using a conventional phenol-chloroform protocol (Sambrook et al., 1989). The purified DNA was resuspended in Tris-EDTA buffer (TE; 10 mM Tris–HCl, 1 mM EDTA, pH 7.5). For RNA isolation, a pool of several frozen whole bodies of adult individuals were mortar-pulverized in liquid nitrogen. The purified RNA was extracted in lysis buffer, containing 35 mM EDTA, 0.7 M LiCl, 7.0% SDS and 200 mM Tris–Cl (pH 9.0), following the protocol by Woo et al. (2005). The purified RNA was eluted in DEPC-treated water and stored at −20°C. DNA quality was assessed using Nanodrop, 1% agarose gels, Qubit fluorometer and the Qubit HS DNA assay reagents. The RNA integrity was assessed using Nanodrop and an Agilent 2100 Bioanalyzer electrophoresis system (Agilent, Santa Clara, CA, USA).

### Paired-End and Mate Pair DNA Fragment Library Construction

The TruSeq DNA Sample Prep kit (Illumina) was used to prepare two paired-end (PE) libraries with insert size 350 bp. In addition, using the Nextera Mate Pair (MP) Sample Preparation kit (Illumina) was used to prepare four MP libraries with insert sizes 3, 5, 8, and 10 kb. We generated a total of 558,951,044 (140 Gbp) PE reads of average length 251 bp and 2,252,642,722 (227 Gbp) MP reads of average length 101 bp (Supplementary Table 1). Ready-to-sequence Illumina libraries were quantified by qPCR using the SYBR Green PCR Master Mix (Applied Biosystems), and library profiles were evaluated with an Agilent 2100 Bioanalyzer (Agilent Technologies, Santa Clara, CA, USA).

### RNA Short Fragment Sequencing (RNA-Seq) and PacBio Isoform Sequencing (Iso-Seq)

For short fragment sequencing, the TruSeq mRNA Prep kit (Illumina) was used to prepare a PE library from total mRNA, which was subsequently sequenced on an Illumina HiSeq 2500. We generated a total of 111,761,580 (11 Gbp) PE reads of length 101 bp (Supplementary Table 1).

For long fragment sequencing, three sequencing libraries (1–2, 2–3, and 3–6 kb) were prepared from polyA+ RNAs according to the PacBio Iso-seq protocol. A total of six Single-Molecule Real-Time cells were run on a PacBio RS II system by DNALink Co. A total of 483,728 reads (1.2 Gbp) were assembled to 110,855 high-quality transcripts (252 Mbp) (Supplementary Table 2).

### k-mer Distribution and Genome Size Estimation

To estimating the genome size, raw PE reads were processed by removing leading and trailing low-quality regions or those that contained the TruSeq index and universal adapters using Trimmomatic (Bolger et al., 2014) v0.36. A 17-mer distribution was generated using JELLYFISH (Marçais and Kingsford, 2011) v2.2.6 and the genome size of *P. hallaensis* was subsequently estimated at 1.43 Gbp using GenomeScope (Vurture et al., 2017) v1.0 where the main peak lied at the k-mer depth of 38 (Supplementary Figure 1).

### Genome Assembly

**Figure 1:**
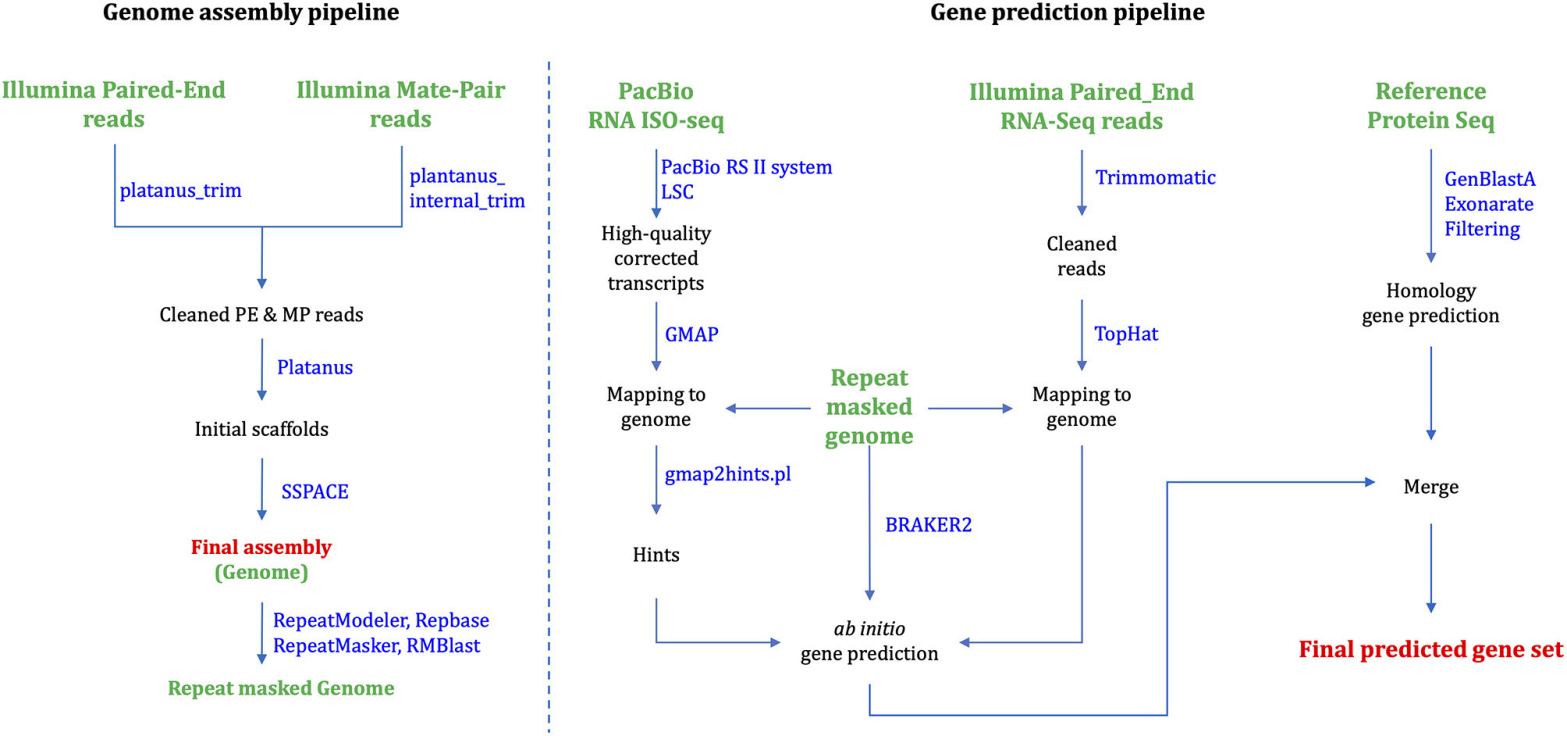
Assembly and gene prediction workflow.

Platanus_trim and Plantanus_internal_trim v1.0.7 trimmed adapters, low-quality reads and uncalled bases from PE and MP raw reads, respectively. Platanus (Kajitani et al., 2014) v1.2.4 assembled the cleaned reads based on automatically optimized multiple k-mer values. We executed individual commands “assemble,” “scaffold,” and “gap_close” in the Platanus assembler suite, successively. We assigned the maximum memory usages as 2,048G for the “assemble” stage, but all the other stages were executed with default options. SSPACE (Boetzer et al., 2010) v3.0 was used for rescaffolding scaffolds larger than 1,000 bp in length using trimmed PE and MP reads in Figure 1. QUAST (Gurevich et al., 2013) v4.5 accessed the length statistics of the genome assembly. The total assembly length is 1.18 Gb, which corresponds to 82.5% of the estimated genome size. The final N50 scaffold is 86.5 kb (Table 1).

**Table 1:** Statistics of the genome assembly.

	**Platanus**	**SSPACE**	**NCBI**
Scaffolds	5,739,039	39,877	39,873
Scaffolds (>1,000)	108,362	39,877	39,873
Total length	1,999,865,159	1,178,051,579	1,177,993,560
Total length (>10,00)	1,079,547,176	1,178,051,579	1,177,993,560
Maximum length	717,908	1,338,718	1,338,718
N50	32,439	86,525	86,525
Gap	19,001,919	117,479„487	117,463,605

The initial assembly was generated by Platanus and rescaffolded by SSPACE. Four scaffolds were removed during NCBI submission process.

### Repeat Annotation

Repetitive elements were annotated as follows. First, tandem repeats were identified using the Tandem Repeats Finder (Benson, 1999) v4.0.7. Next, transposable elements (TEs) were identified by *de novo* [RepeatModeler (Abrusán et al., 2009) v1.0.10] and homology-based approaches [Repbase (Jurka et al., 2005) v4.0.7, RepeatMasker (Bedell et al., 2000) v4.0.7 and RMBlast (Bedell et al., 2000) v2.2.27+]. All TEs were merged and accounted for 33.71% of the genome, with unknown repeats ranking the largest portion (16.84%) (Supplementary Table 3).

### Gene Prediction and Annotation

To predict protein-coding genes, we combined *ab initio* and homology-based gene prediction methods (Figure 1). For the *ab initio* gene prediction, two hint files were generated from an Illumina RNA-seq and PacBio Iso-seq. RNA-seq reads were aligned to the repeat-masked genome assembly using Tophat (Kim et al., 2013) v2.1.1. Iso-seq was proceeded to obtain intron hints, as described in Minoche et al. (2015): (1) run LSC (Au et al., 2012) v2.0 to correct errors for full-length transcripts, (2) align the corrected transcripts to the genome using GMAP (Wu and Watanabe, 2005) 2019-06-10, and (3) generate intron hints from aligned sequences using blat2hints.pl v3.3.2 in the AUGUSTUS package. We obtained 119,797 and 351,813 hints from RNA-seq and Iso-seq, respectively. BRAKER (Hoff et al., 2016) v2.0 predicted 104,121 genes, which incorporated outputs from GeneMark-ET (Lomsadze et al., 2014) v4.38 and AUGUSTUS (Stanke et al., 2008) v3.3.3. GeneMark-ET predicts genes with unsupervised training, whereas AUGUSTUS predicts genes with supervised training based on intron and protein hints. Finally, we obtained a total of 16,648 protein-coding genes for *ab initio* prediction (Table 2).

**Table 2:** Statistics of predicted protein-coding genes.

	**Number of genes**	**Number of transcripts**	**Number of non-overlapping exons**	**Number of non-overlapping introns**	**Average number of exons**	**Number of alternatively spliced genes**
*De novo*	16,648	18,424	119,631	104,504	7.4	1,450
Homology	12,899	12,899	62,135	49,056	4.9	0
	3,132	3,132	7,768	4,628	2.5	0
Merged	19,780	21,556	127,364	109,129	6.7	1,450
	**Number of single exon transcripts**	**Average gene length (bp)**	**Average transcript length (bp)**		**Average exon length (bp)**	**Average intron length (bp)**
			**w/introns**	**w/o introns**		
*De novo*	0	12,892.7	13,627.1	1,565.6	211.0	1,863.6
Homology	2,685	8,738.3	8,738.3	1,027.1	211.9	2,010.6
	1,729	4,395.0	4,395.0	761.6	306.4	2,450.4
Merged	1,729	11,547.1	12,285.8	1,448.8	216.9	1,888.5

Homology-based method predicted 12,899 genes, of which 3,132 genes were merged to the final genes.

For the homology gene predictions, we aligned the assembly of *P. hallaensis* against the genes of *Daphnia pulex, Drosophila melanogaster, Eulimnadia texana, Folsomia candida, Hyalella azteca, Lepeophtheirus salmonis, Oithona nana, Parasteatoda tepidariorum, Parhyale hawaiensis, Strigamia maritima, Tigriopus kingsejongensis*, and arthropoda in orthoDB v9 using TBLASTN (Camacho et al., 2009) v2.2.18 with an E-value cutoff of 1E-5. GenBlastA (She et al., 2009) v1.0.4 was used to cluster matching sequences, and retain only best-matched regions. Then, Exonerate (Slater and Birney, 2005) v2.2.0 predicted gene models. As a result, we obtained a total of 12,899 genes using a homology-based approach (Table 2).

Finally, the two outputs were combined by placing homology predictions to *ab initio* prediction only when there is no conflict. Then we removed the predicted coding sequences (CDSs) if those contain premature stop codons or those were not supported by hints. As a result, 19,780 protein-coding genes were predicted for the draft assembly of *P. hallaensis* (Table 2). The predicted genes were annotated using InterProScan (Jones et al., 2014) v5.16-55.0 with various databases, including Hamap (Lima et al., 2008), Pfam (Punta et al., 2011), PIRSF (Nikolskaya et al., 2006), PRINTS (Attwood et al., 2000), ProDom (Bru et al., 2005), PROSITE (Sigrist et al., 2009), SUPERFAMILY (Madera et al., 2004), and TIGRFAM (Haft et al., 2012).

### Genome Assembly and Gene Prediction Quality Assessment

BUSCO (Simão et al., 2015) v3.0.2 evaluated genome completeness with Arthropoda conserved genes databases. The complete BUSCO value of the genome assembly was 84.7% while those of predicted genes was higher (86.6%) (Supplementary Table 4). Three bacterial scaffolds and one adapter contaminated scaffold were detected and removed during NCBI submission process (Table 1).

### Comparison With Other Arthropod Genomes

An extensive comparison of orthologous genes among 13 arthropod genomes (*P. hallaensis, D. pulex, D. melanogaster, E. texana, F. candida, H*. *azteca, L. salmonis, O. nana, P. tepidariorum, P*. *hawaiensis, S. maritima, T. kingsejongensis*, and *T*. *longiramus*; Supplementary Table 5) was performed using OrthoMCL (Li et al., 2003) v2.0.9. We obtained 3,843 unique gene clusters in *P. hallaensis*. GO term analysis was performed using Fisher's exact test followed by false discovery rate correction to identify functionally enriched GO terms among the unique genes relative to the “genome background,” as annotated by Pfam. The GO terms with *q* < 0.05 were responsible for oxidoreductase activity, ion binding, nervous system process, transferase activity, transferring glycosyl groups, and GTPase activity (Supplementary File 1).

Supplementary Figure 2 shows a Venn diagram of orthologous gene clusters among the 3 closely related species to *P. hallaensis*. While all species had 8,498 common clusters, *H. azteca, P. hawaiensis, P. hallaensis*, and *T*. *longiramus* had 3,748, 10,741, 4,010, and 3,134 unique clusters, respectively.

**Figure 2:**
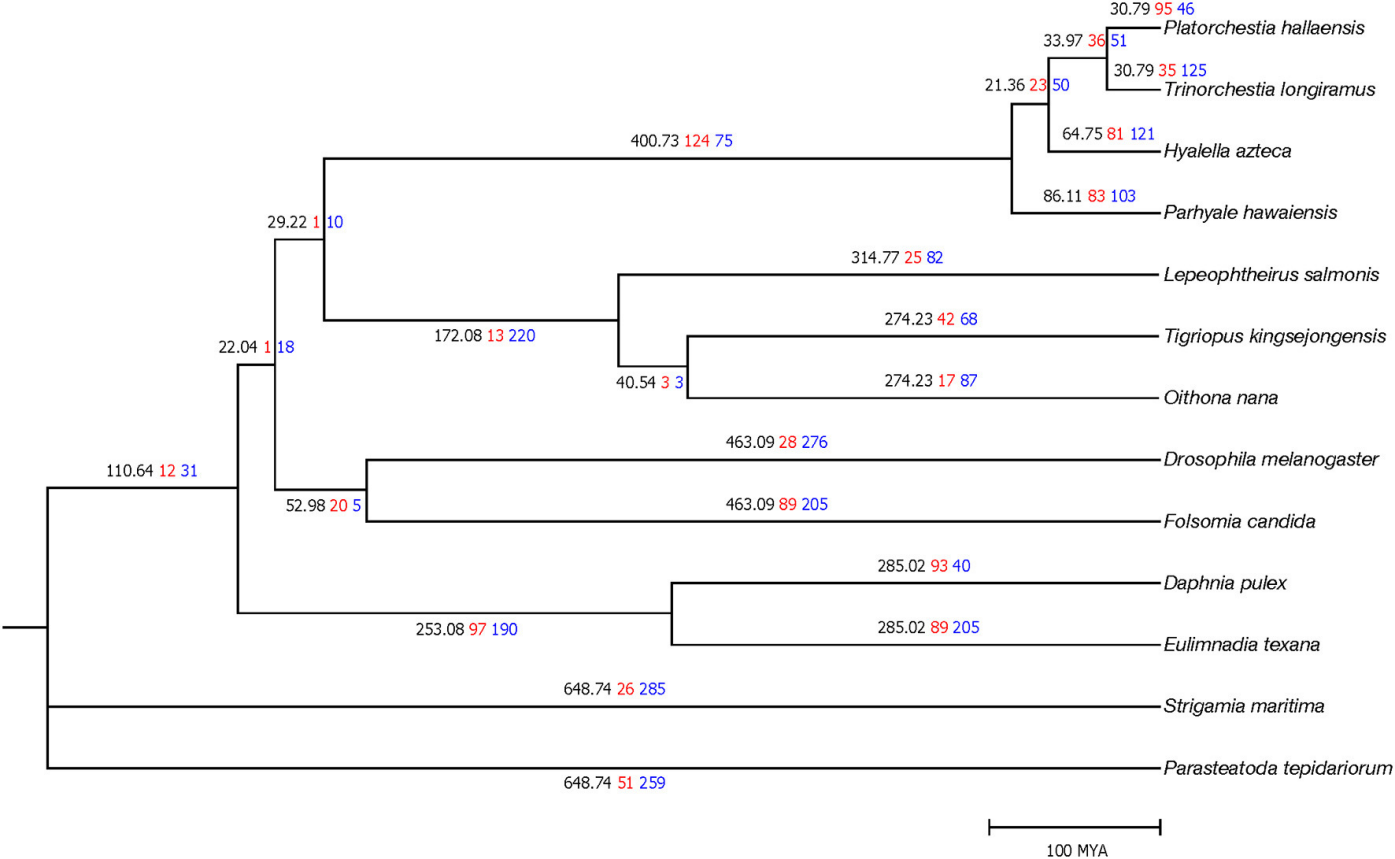
Time tree of the 13 arthropod species where the first (black) numbers represent divergence time as well as the second (red) and third (blue) numbers represent the number of orthologous gene clusters expanded and contracted, respectively. Divergence time is scaled in millions of years.

MUSCLE (Edgar, 2004) v3.8.31 aligned 378 single-copy protein sequences after orthologous gene clustering. trimAl (Capella-Gutiérrez et al., 2009) v3.1.1 filtered low alignment quality regions. RAxML (Stamatakis, 2014) v8.2.10 constructed a phylogenetic tree with the PROTGAMMAJTT model (100 bootstrap replicates). MEGA7 (Kumar et al., 2016) v7.00 calculated divergence time with the Jones–Taylor–Thornton model and the previously determined topology. The TimeTree database (Hedges et al., 2006) was used to take calibration times of *Folsomia*–*Drosophila* divergence (442–496 MYA) and *Eulimnadia-Daphnia* divergence (128–298 MYA). *P. hallaensis* diverged from *T*. *longiramus* and *H*. *azteca* during the Middle and Early Cenozoic era, ~29 and 60 million years ago, respectively (Figure 2).

CAFE (Han et al., 2013) v4.0 conducted a gene expansion and contraction analysis with the identified orthologous gene clusters and the estimated phylogenetic information. Of *P. hallaensis*, 95 and 46 orthologous gene clusters were expanded and contracted, respectively, with respect to its common ancestor with *T. longiramus* (Figure 2). The expanded clusters were associated with multidrug resistance, glycoprotein, heat shock, glucuronosyl transfer, glucose regulation, cytochrome, Xanthine dehydrogenase, notch, ubiquitin-protein ligase, NADH dehydrogenase, murinoglobulin, zinc finger transcription factor, sodium-coupled monocarboxylate transporter, histone-lysine N-methyltransferase, chorion peroxidase, facilitated trehalose transporter, salivary glue, endoglucanase, glucosylceramidase, alpha-L-fucosidase, macrophage mannose receptor, lysozyme C-1, formaldehyde dehydrogenase, down syndrome cell adhesion, methionine synthase, sortilin-related receptor, CD209 antigen, prolow-density lipoprotein receptor, Cathepsin, Zinc finger protein, and RING-H2 finger protein (Supplementary File 2). The contracted clusters were associated with histone H2A, glutamate receptor, Glucose dehydrogenase, vulva defective, collagen alpha-2, antileukoproteinase, and TATA element modulatory factor (Supplementary File 2).

## Usage Notes

All analyses were conducted on Linux systems, and used parameters are given in Supplementary Table 6. It shows the software versions, settings, and parameters. If not mentioned otherwise, the command line at each step was executed using default settings.

## Data Availability Statement

The datasets presented in this study can be found in online repositories. The names of the repository/repositories and accession number(s) can be found at: https://www.ncbi.nlm.nih.gov/, ASM1422093v1; https://www.ncbi.nlm.nih.gov/, PRJNA645242.

## Author Contributions

J-HC, YY, and MY conceived concept. TJ, MK, and MY provided the sample. J-HC and YY designed the experiments. AP, OC, JY, SB, YY, and J-HC analyzed the genomic data. SB and YY deposited the data into NCBI. AP, JY, SB, MY, YY, and J-HC wrote the paper. All authors reviewed the manuscript.

## Conflict of Interest

OC was employed by company Clinomics Inc. The remaining authors declare that the research was conducted in the absence of any commercial or financial relationships that could be construed as a potential conflict of interest.

## Supplementary Material

Supplementary Figure 1 Genome size estimation by k-mer distribution.

Supplementary Figure 2 A Venn diagram of unique and shared orthologous gene clusters in 4 Talitroidea species: *Platorchestia hallaensis, Parhyale hawaiensis, Hyalella azteca* and *Trinorchestia longiramus*.
