# Supplementary figures and images for: The Draft Genome Sequence of a New Land-Hopper Platorchestia hallaensis
Source: Front Genet. 2021 Jan 11;11:621301. doi: 10.3389/fgene.2020.621301 (PMC7831040; doi:10.3389/fgene.2020.621301)

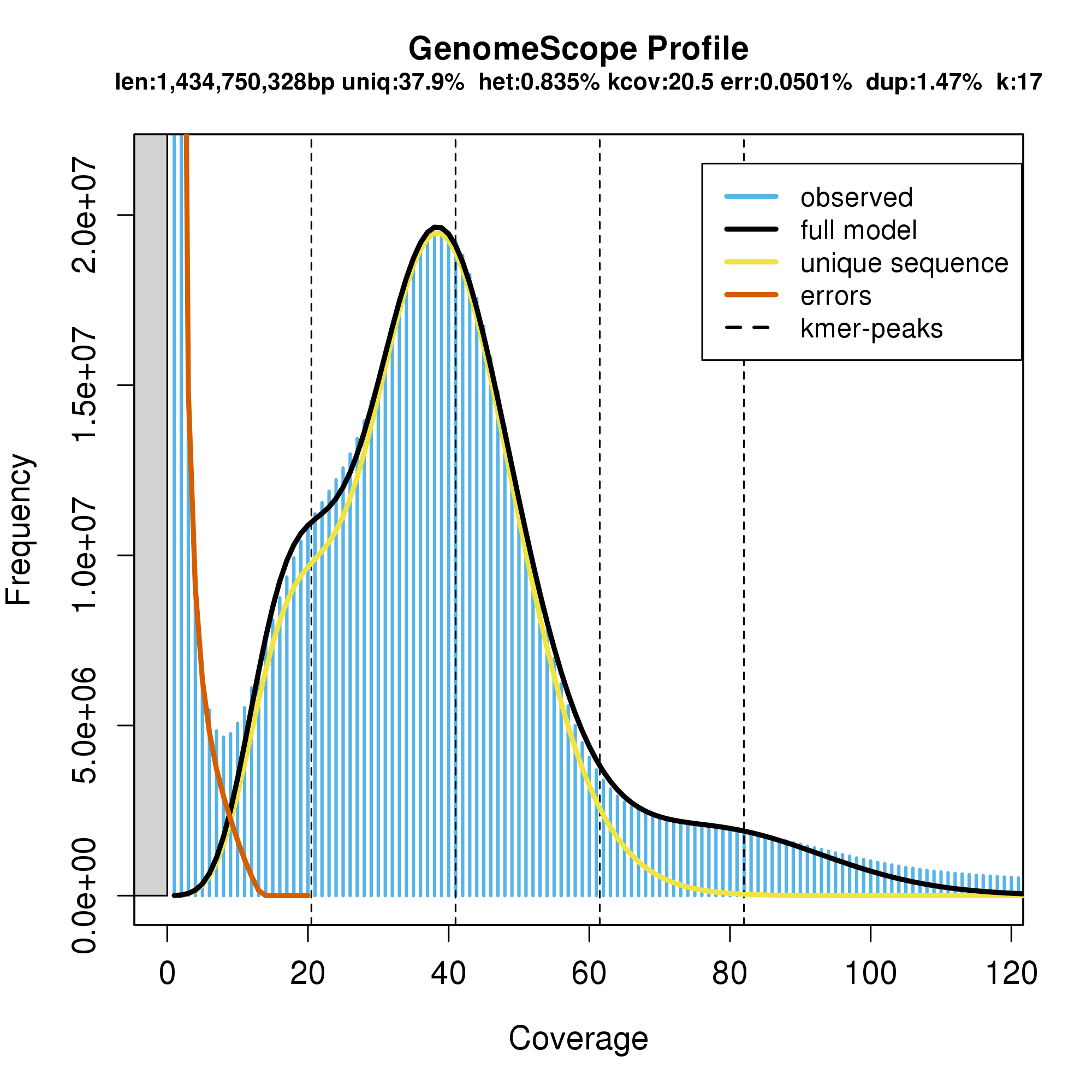

Supplement: Supplementary Figure 1 — Genome size estimation by k-mer distribution. [file Image_1.PNG]

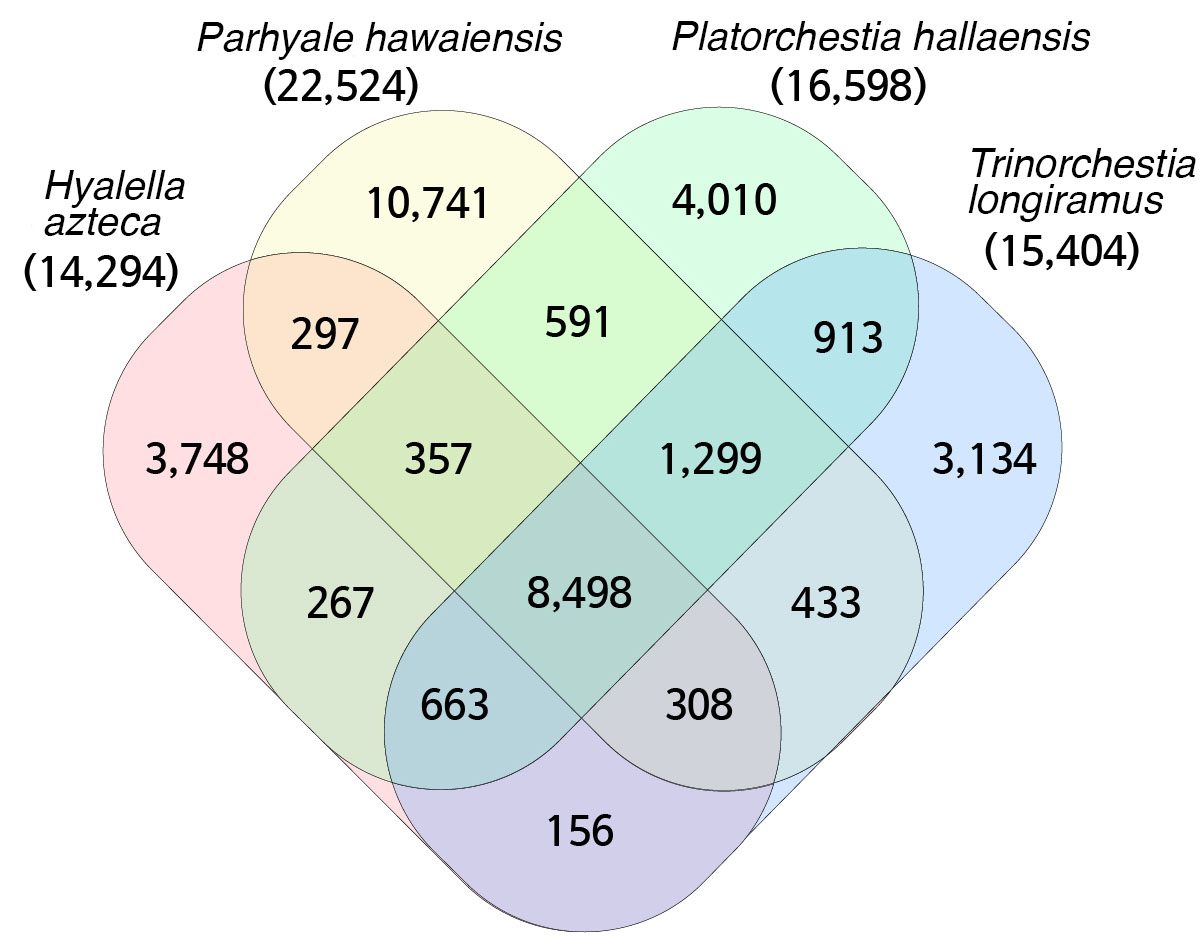

Supplement: Supplementary Figure 2 — A Venn diagram of unique and shared orthologous gene clusters in 4 Talitroidea species: Platorchestia hallaensis, Parhyale hawaiensis, Hyalella azteca and Trinorchestia longiramus. [file Image_2.JPEG]
