# Supplementary material for: The Draft Genome Sequence of a New Land-Hopper Platorchestia hallaensis
Source: Front Genet. 2021 Jan 11;11:621301. doi: 10.3389/fgene.2020.621301 (PMC7831040; doi:10.3389/fgene.2020.621301)
Supplement: Supplementary file 3 [file Table_1.docx]

**Supplementary Table 1.** Sequence libraries and data yield from Illumina DNA and RNA sequencing.

|  | Library type | Insert Size (bp) | Read Length (bp) | Raw bases (Gbp) | Raw reads | SRA accessions |
| --- | --- | --- | --- | --- | --- | --- |
| DNA | Paired-end (PE) | 350 | 251 | 70.148 | 279,475,522 | SRR12464061 |
|  |  | 350 | 251 | 70.148 | 279,475,522 |  |
|  | Total |  |  | 140.296 | 558,951,044 |  |
|  | Mate-pair (MP) | 3K | 101 | 29.016 | 287,288,554 | SRR12464061 |
|  |  | 3K | 101 | 29.016 | 287,288,554 |  |
|  |  | 5K | 101 | 27.747 | 274,725,051 | SRR12464064 |
|  |  | 5K | 101 | 27.747 | 274,725,051 |  |
|  |  | 8K | 101 | 28.876 | 285,896,853 | SRR12464063 |
|  |  | 8K | 101 | 28.876 | 285,896,853 |  |
|  |  | 10K | 101 | 28.120 | 278,410,903 | SRR12464062 |
|  |  | 10K | 101 | 28.120 | 278,410,903 |  |
|  | Total |  |  | 227.517 | 2,252,642,722 |  |
| RNA | PE | 140 | 101 | 5.643 | 55,880,790 | SRR12464060 |
|  |  | 140 | 101 | 5.643 | 55,880,790 |  |
|  | Total |  |  | 11.286 | 111,761,580 |  |
