# Supplementary material for: The Draft Genome Sequence of a New Land-Hopper Platorchestia hallaensis
Source: Front Genet. 2021 Jan 11;11:621301. doi: 10.3389/fgene.2020.621301 (PMC7831040; doi:10.3389/fgene.2020.621301)
Supplement: Supplementary file 4 [file Table_2.docx]

**Supplementary Table 2**. Sequencing libraries and data yields from PacBio RNA sequencing.

| Library size (Kb) | Average read Length (bp) | Raw bases (Gbp) | Raw reads | Polished high-quality isoforms | Polished high-quality bases (Mbp) | SRA accession |
| --- | --- | --- | --- | --- | --- | --- |
| 1-2 | 1,584 | 0.127 | 80,371 | 110,855 | 252 | SRR12480621 |
|  | 1,651 | 0.127 | 77,020 |  |  | SRR12480618 |
| 2-3 | 2,531 | 0.216 | 85,162 |  |  | SRR12480616 |
|  | 2,762 | 0.248 | 89,611 |  |  | SRR12480619 |
| 3-6 | 3,495 | 0.296 | 84,802 |  |  | SRR12480620 |
|  | 3,272 | 0.218 | 66,762 |  |  | SRR12480617 |
| Total | 2,549 | 1.233 | 483,728 |  |  |  |
