# Supplementary material for: The Draft Genome Sequence of a New Land-Hopper Platorchestia hallaensis
Source: Front Genet. 2021 Jan 11;11:621301. doi: 10.3389/fgene.2020.621301 (PMC7831040; doi:10.3389/fgene.2020.621301)
Supplement: Supplementary file 5 [file Table_3.docx]

**Supplementary Table 3**. Statistics of repetitive elements.

|  | Total (bp) | % of genome |
| --- | --- | --- |
| DNA | 58,289,912 | 4.95 |
| LINE | 53,947,304 | 4.58 |
| LTR | 19,146,427 | 1.63 |
| Low complexity | 1,202,626 | 0.14 |
| SINE | 2,035,647 | 0.17 |
| Satellite | 1,016,423 | 0.09 |
| Simple repeat | 24,129,736 | 2.05 |
| Tandem Repeat | 74,841,768 | 6.35 |
| Unknown | 198,327,427 | 16.84 |
| Unspecified | 5,335,641 | 0.45 |
| Non-overlapping total | 397,171,404 | 33.71 |
