# Supplementary material for: The Draft Genome Sequence of a New Land-Hopper Platorchestia hallaensis
Source: Front Genet. 2021 Jan 11;11:621301. doi: 10.3389/fgene.2020.621301 (PMC7831040; doi:10.3389/fgene.2020.621301)
Supplement: Supplementary file 6 [file Table_4.docx]

**Supplementary Table 4**. BUSCO assessment of genome assembly and gene prediction.

| Genome assembly | Number of  Scaffolds | BUSCO (Arthropoda) |
| --- | --- | --- |
| Platanus | 5,739,039 | C:77.8%[S:76.7%,D:1.1%],F:12.8%,M:9.4%,n:1066 |
| SSPACE | 39,877 | C:84.7%[S:84.0%,D:0.7%],F:7.0%,M:8.3%,n:1066 |
| Gene prediction | Number of Genes |  |
| Merged | 19,780 | C:86.6%[S:77.7%,D:8.9%],F:10.1%,M:3.3%,n:1066 |
