# Supplementary material for: The Draft Genome Sequence of a New Land-Hopper Platorchestia hallaensis
Source: Front Genet. 2021 Jan 11;11:621301. doi: 10.3389/fgene.2020.621301 (PMC7831040; doi:10.3389/fgene.2020.621301)
Supplement: Supplementary file 7 [file Table_5.docx]

**Supplementary Table 5**. Statistics of 13 arthropod genomes.

| Species | # proteins | # gene clusters | Avg. # copies |
| --- | --- | --- | --- |
| *Daphnia pulex* | 30,141 | 17,407 | 1.7 |
| *Drosophila melanogaster* | 13,871 | 10,894 | 1.3 |
| *Eulimnadia texana* | 17,475 | 12,876 | 1.4 |
| *Folsomia candida* | 28,732 | 13,745 | 2.1 |
| *Hyalella azteca* | 17,509 | 14,294 | 1.2 |
| *Lepeophtheirus salmonis* | 13,081 | 11,291 | 1.2 |
| *Oithona nana* | 13,882 | 12,262 | 1.1 |
| *Parasteatoda tepidariorum* | 16,574 | 10,854 | 1.5 |
| *Parhyale hawaiensis* | 28,617 | 22,524 | 1.3 |
| *Platorchestia hallaensis* | 19,780 | 16,598 | 1.3 |
| *Strigamia maritima* | 14,930 | 11,233 | 1.3 |
| *Tigriopus kingsejongensis* | 12,772 | 10,044 | 1.3 |
| *Trinorchestia longiramus* | 19,692 | 15,404 | 1.3 |
