# Supplementary material for: The Draft Genome Sequence of a New Land-Hopper Platorchestia hallaensis
Source: Front Genet. 2021 Jan 11;11:621301. doi: 10.3389/fgene.2020.621301 (PMC7831040; doi:10.3389/fgene.2020.621301)
Supplement: Supplementary file 8 [file Table_6.docx]

**Supplementary Table 6.** A list of software and parameters used for genome analysis.

| **Softwares** | **Version** | **Parameters/Commands** |
| --- | --- | --- |
| Trimmomatic | 0.36 | -phred33 ILLUMINACLIP:adapter:2:30:10 LEADING:3 TRAILING:3 SLIDINGWINDOW:4:15 MINLEN:36 |
| JELLYFISH | 2.2.6 | -C -m 17 |
| GenomeScope | 1.0 | 17 251 |
| Platanus trim | 1.0.7 | platanus_trim (for PE reads), platanus_internal_trim (for MP reads) |
| Platanus | 1.2.4 | step-1: assemble -m 2048, step-2: scaffold, step-3: gap_close |
| SSPACE Standard | 3.0 | default |
| QUAST | 4.5 | default |
| BUSCO | 3.0.2 | -l arthropoda_odb9 |
| TandemRepeatFinder | 4.0.7 | 2 7 7 80 10 50 500 -f -d -m -h |
| RepeatModeler | 1.0.10 | -engine ncbi -pa 4 |
| RepeatMasker (*de novo*) | 4.0.7 | -lib Library.fa -e ncbi -pa 16 –gccalc –poly -gff |
| RepeatMasker (homology) | 4.0.7 | -species drosophila -e ncbi -pa 16 –gccalc –poly -gff |
| LSC | 2.0 | default |
| GMAP | 2019-06-10 | -B 5 |
| gmap2hints.sh | 3.3.2 | --intronsonly --priority=3 --nomult --ep_cutoff=20 |
| Tophat | 2.1.1 | --microexon-search --mate-std-dev 26 --mate-inner-dist 38 --min-intron-length 30 --min-coverage-intron 30 --min-segment-intron 30 |
| GenBlastA | 1.0.4 | -p T -e 1e-5 -g T -f F -a 0.5 -d 100000 -r 100 -c 0.01 -s -100 |
| Exonerate | 2.2.0 | --model protein2genome --percent 30 --showvulgar no --showalignment yes --showquerygff no --showtargetgff yes --targetchunkid 1 --targetchunktotal 100 |
| BRAKER | 2.0 | --species=*Platorchestia* --useexisting --AUGUSTUS_CONFIG_PATH=augustus-3.3.3/config --AUGUSTUS_BIN_PATH=augustus-3.3.2/bin --AUGUSTUS_SCRIPTS_PATH=augustus-3.3.2/scripts --GENEMARK_PATH=gm_et-2019/gmes_petap --cores=48 --AUGUSTUS_ab_initio --gff3 --alternatives-from-evidence=true --softmasking --species=Platorchestia --workingdir=. --genome=scaffolds.fa --bam=tophat/accepted_hits.bam --hints=isoseq/full_LR.gff |
| InterProscan | 5.16-55.0 | -appl HAMAP,ProDom,PRINTS,Pfam,TIGRFAM,SUPERFAMILY,ProSitePatterns,ProSiteProfiles -goterms -iprlookup |
| OrthoMCL | 2.0.9 | -I 1.5 |
| MUSCLE | 3.8.31 | default |
| trimAl | 3.1.1 | -gappyout -phylip |
| RAxML | 8.2.10 | -m PROTGAMMAJTT |
| MEGA | 7.00 | megacc |
| CAFÉ | 4.0 | default |
